# PITX2 in pancreatic stellate cells promotes EMT in pancreatic cancer cells via the Wnt/β-catenin pathway: PITX2 promotes pancreatic cancer progression via Wnt/β-catenin pathway

**DOI:** 10.3724/abbs.2023118

**Published:** 2023-06-19

**Authors:** Di Wu, Weibo Chen, Yang Yang, Yi Qin, Guangchen Zu, Yue Zhang, Yong An, Donglin Sun, Xiaowu Xu, Xuemin Chen

**Affiliations:** 1 Department of Hepatopancreatobiliary Third Affiliated Hospital of Soochow University Changzhou 213001 China; 2 Department of Pancreatic Surgery Fudan University Shanghai Cancer Center Shanghai 200032 China

**Keywords:** pancreatic stellate cell, PITX2, pancreatic cancer, EMT, Wnt/β-catenin pathway

## Abstract

Since the prognosis of patients with pancreatic cancer is very poor and there is a lack of treatment methods, this study is performed to investigate the function of PITX2 in pancreatic stellate cells (PSCs) in the progression of pancreatic cancer. Scientific hypotheses are proposed according to bioinformatics analysis and tissue microarray analysis. Stable knockdown of *PITX2* in PSCs is achieved through lentiviral infection. The relative expressions of PITX2, α-SMA, vimentin, CTNNB1, AXIN1 and LEF1 are measured in wild-type PSCs and *PITX2*-knockdown PSCs. Proliferative capacity is measured by EdU assay. After coculture with PSCs, the proliferation, invasion and migration capacity of pancreatic cancer cells are tested. EMT and Wnt/β-catenin downstream genes of pancreatic cancer cells are investigated to reveal the potential mechanism. Bioinformatics analysis reveals that the *PITX2* gene is highly expressed in stromal cells in pancreatic cancer and is correlated with squamous-type PDAC. Analysis of PDAC tissue microarray further demonstrates that high PITX2 level in stromal cells is correlated with poor prognosis in PDAC. After stable knockdown of *PITX2* in PSCs, the relative protein levels of α-SMA, vimentin, CTNNB1, AXIN1 and LEF1 are decreased, and the proliferative capacity of PSCs is also decreased. After coculture with PSCs, in which PITX2 expression is downregulated, the proliferation, invasion and migration capacities of pancreatic cancer cells are inhibited. Thus, our results show that *PITX2*-silenced PSCs inhibit the growth, migration and invasion of pancreatic cancer cells via reduced EMT and Wnt/β-catenin signaling.

## Introduction

Patients with pancreatic ductal adenocarcinoma (PDAC) urgently need effective treatment. The prognosis of PDAC patients is very poor. In the USA, the 5-year survival rate is only 10% [1]. The incidence of PDAC is also increasing and is expected to become the second leading cause of cancer-related deaths by 2030 [2]. Inefficient diagnosis and strong drug resistance are the main causes of poor prognosis.

The tumor microenvironment (TME) has received increasing attention for its potential function in cancer cell progression [ 3‒ 5]. Pancreatic cancer has a dense extracellular matrix, which plays an important role in tumor development and chemotherapy tolerance. In pancreatic cancer, activated pancreatic stellate cells (PSCs) together with a large amount of extracellular matrix secreted by PSCs constitute an important component of the tumor microenvironment.

*PITX* genes belong to the PRD class of homeobox genes that are highly conserved. Vertebrates possess three *PITX* paralogues, *PITX1* , *PITX2* and *PITX3*, while invertebrates have only one *PITX* gene. PITX2 regulates pituitary, facial, dental, cardiac, intestinal and facial, and skeletal muscle development. Recently, the involvement of the *PITX2* gene in tumorigenesis has made it be a diagnostic marker and potential drug target [6]. Previous studies reported that elevated expression of PITX2 is associated with advanced progression and poor prognosis of lung adenocarcinoma [ 7, 8], colorectal cancer [ 9, 10], ovarian cancer [11], esophageal squamous cell carcinoma [12] and thyroid cancer [ 13, 14], but few studies have reported the connection between PITX2 and pancreatic cancer.

In the present study, we aimed to investigate the relationship between PITX2 and PDAC to further illuminate the mechanism of tumor progression and provide promising cellular targets to develop therapeutic effects.

## Materials and Methods

### Bioinformatics analysis

Gene expression of patient-derived tumor xenografts (PDXs) was obtained from the supplementary data set of Moffitt’s research [15]. Gene expression data of primary tumors were obtained from GEO data uploaded by Moffitt ( http://www.ncbi.nlm.nih.gov/geo/query/acc.cgiacc=GSE21501). The chip data obtained were analyzed using the GEOquery package of R software and were standardized and log converted. Other PDX data were obtained from the website published by Nicole ( https://github.com/RemyNicolle/PaCaOmicsDATA/blob/master/RNA/), and the raw count data of tumor epithelial cells and stromal cells were downloaded. The edgeR method was used to standardize the samples, and the voom function in the Limma package of R software was used to convert the raw count data to the logCPM value. Mouse Genome Informatics ( http://www.informatics.jax.org/) was used to integrate the expression data of human (tumor) and mouse (matrix) in Nicole’s PDX model based on the information of homologous genes of human and mouse genes. The Limma package of R software was used to carry out the paired difference test between stromal cells and tumor cells. FDR was used for multiple inspection and correction. *P*<0.05 was regarded as significant difference. After analyzing the data of differentially expressed genes, we take log2 (fold change) as the abscissa and the negative logarithm of FDR-log10 (FDR) as the ordinate to obtain the Volcano Plot. The characteristic genes of two molecular subtypes of PDAC (classic and squamous subtypes) in the article published by Yue *et al*. [16] were extracted. Next, we used the characteristic genes of two molecular subtypes of PDAC as gene sets to conduct gene set enrichment analysis (GSEA).

### Immunofluorescence staining and tissue microarray analysis

Immunofluorescence staining and tissue microarray analysis were performed by Shanghai Weiao Biotechnology Co., Ltd. (Shanghai, China). A paired *t* test was conducted to compare PITX2 expression in parenchymal cells and mesenchymal cells. The survival curve was mapped with PITX2 expression in parenchymal cells equal to 0.5 as the cut-off value. Finally, univariate analysis and multivariate logistic regression analysis were conducted to identify possible factors affecting prognosis.

### Pancreatic stellate cell (PSC) isolation and culture

Primary human PSCs were isolated from resected pancreatic tumors. Briefly, freshly resected pancreatic tumor tissue was dissected into 1-mm ^3^ pieces, plated in uncoated wells with DMEM (Gibco, Carlsbad, USA) supplemented with 10% FBS (Gibco), 2 ng/mL human EGF (Abcam, Cambridge, UK) and 1% antibiotics (Gibco), and incubated for 2 weeks to allow for PSC outgrowth. The protocols used in the present study were approved by the Ethics Committee of the Third Affiliated Hospital of Soochow University and conducted in full accordance with ethical principles.

### Cell culture and reagents

The PDAC cell line (SW1990) was purchased from BeNa Culture Collection (Beijing, China). PSCs were obtained from resected pancreatic tumors. Both cell lines were cultured in DMEM supplemented with 10% fetal bovine serum. All cells were cultured at 37°C in a humidified incubator containing 5% CO _2_.

### Cellular immunofluorescence

After fixation with polyformaldehyde for half an hour, samples were blocked with 5% BSA and treated with 0.1% Triton X-100 to increase cell membrane permeability. Then, the samples were incubated with primary antibodies, including anti-α-SMA antibody (ab124964; 1:500; Abcam), anti-vimentin antibody (ab8978; 1:1000; Abcam), anti-PITX2 antibody (ab98297; 1:1000; Abcam), and anti-α-SMA antibody (MA1-06110; 1:200; Invitrogen, Carlsbad, USA) for 12 h at 4°C. Then, anti-mouse IgG (H+L)-Alexa Fluor 488 (ab150113; Abcam), anti-rabbit IgG (H+L)-Alexa Fluor 647 (ab150079; Abcam), anti-mouse IgG (H+L)-Alexa Fluor 647 (ab150115; Abcam), and anti-rabbit IgG (H+L)-Alexa Fluor 488 (ab150077; Abcam) secondary antibodies were used at a dilution of 1:400. Photos were taken with a fluorescence microscope (Leica, Solms, Germany).

### Stable knockdown of *PITX2* by lentiviral vectors

Stable knockdown of *PITX2* was induced through lentiviral infection, and lentiviral plasmids encoding PITX2 (ShPITX2 ^1#^ and ShPITX2 ^2#^) and a negative control (NC) were constructed by HANBIO (Shanghai, China). The sequences of shRNAs are shown as the follows: ShPITX2 ^1#^: 5′-GCTGTGTGGACCAACCTTA-3′; ShPITX2 ^2#^: 5′-CCAACTCTATCTCGTCCAT-3′; and NC: 5′-TTCTCCGAACGTGTCACGT-3′. PITX2 interference efficiency in PSCs was determined by western blot analysis. After infection, the stably infected cells were selected by puromycin for further use.

### Western blot analysis

Western blot analysis was conducted as previously reported [17]. The signals were developed by an ECL system (Millipore, Billerica, USA) and captured by a Tanon 5200 Chemiluminescent Imaging System (Shanghai, China). The antibodies used were as follows: anti-PITX2 antibody (ab98297; 1:1000; Abcam), anti-α-SMA antibody (ab124964; 1:1000; Abcam), anti-vimentin antibody (ab8978; 1:1000; Abcam), anti-CTNNB1 antibody(BF8016; 1:500; Affinity, Changzhou, China), anti-LEF1 antibody (DF7570; 1:2000; Affinity), anti-AXIN1 antibody (DF9264; 1:1000; Affinity), anti-β-actin antibody (3700; 1:1000; CST, Danvers, USA), anti-CyclinD1 antibody (2978; 1:1000; CST), anti-CD44 antibody (3570; 1:1000; CST), anti-c-Myc antibody (5605; 1:1000; CST), anti-c-Jun antibody (9165; 1:1000; CST), anti- TCF1/TCF7 antibody (2203; 1:1000; CST), anti- N-Cadherin antibody (13116; 1:1000; CST), anti- E-Cadherin antibody (3195; 1:1000; CST), anti- ZEB1 antibody (3396; 1:1000; CST), anti-vimentin (5741; 1:1000; CST), and anti-Snail (3879; 1:1000; CST). Anti-rabbit IgG (H+L)-HRP (S0001; 1:5000; Affinity) was used as the secondary antibody.

### EdU assay

The EdU assay was performed using the Cell-Light EdU Apollo567 In Vitro Imaging Kit (C10310-1; RiboBio, Guangzhou, China) according to the manufacturer’s instructions. Briefly, PSCs were seeded in 96-well plates at 1×10 ^4^ cells/well. After culture in medium containing 50 μM EdU for 2 h, the cells were washed with PBS and fixed with polyformaldehyde. Triton X-100 (0.5%) was used to increase cell membrane permeability. Cells were incubated with Apollo567, followed by Hoechst staining. Each well was randomly imaged in five fields under a fluorescence microscope (Leica). All images were processed with ImageJ software (National institutes of Health, Bethesda, USA). The proportion of EdU-incorporated cells was calculated.

### Coculture

PANC-1 and SW1990 cells (1×10 ^6^ cells/2.5 mL) were seeded in one of the 6-well plates, while PSCs (5×10 ^5^ cells/1.5 mL) were seeded in the upper transwell chamber (0.4-μm pore size). They were cocultured for 3 days, and PANC-1 and SW1990 cells were harvested for further use.

### Cell proliferation assay

Cell proliferation was assessed using Cell Counting Kit-8 (CCK-8; Sangon Biotech, Shanghai, China). Briefly, cells (100 μL) were plated in 96-well plates at a density of 4000 cells/well, and the absorbance was measured at 450 nm at different time points (0 h, 24 h, 48 h, 72 h, and 96 h) with a microplate reader.

### Invasion and migration assay

The invasiveness and migration capacities of pancreatic cancer cells were assessed by determining the number of cells invading or migrating across transwell chambers. For invasion assays, pancreatic cancer cells (5×10 ^4^ cells/100 μL) were seeded in the upper transwell chamber (8-μm pore size), with its membrane precoated with Matrigel (20 mg/well; BD Biosciences, Bedford, USA). PSCs (1×10 ^5^ cells/600 μL) were seeded in the lower chamber. Thereafter, the cells were incubated for 48 h, and the number of invading pancreatic cancer cells was counted. Cell migration assays were performed using the same protocol as the invasion assay without a Matrigel-coated membrane. Cells were allowed to migrate and were counted 24 h after cell seeding into the upper chamber.

### Flow cytometry assay

Flow cytometry analysis was performed to detect cell cycle distribution. After coculture with PSCs for 3 days, pancreatic cancer cells were harvested and fixed in 1 mL of 70% ethanol at 4°C overnight. The ethanol-suspended cells were centrifuged at 1000 *g* and stained with PI staining solution for 30 min in the dark at 37°C. A flow cytometer (Beckman Coulter, Brea, USA) was used to detect the cell cycle distribution. The percentages of cells in G1, S, and G2/M phases were calculated and compared.

### Statistical analysis

Data are presented as the mean±SEM from at least three independent experiments. Statistical analysis was performed using SPSS Statistics 25. Statistical significance was set at *P*<0.05.

## Results

### *PITX2* gene is highly expressed in stromal cells in pancreatic cancer and correlated with squamous-type PDAC

**Figure 1:**
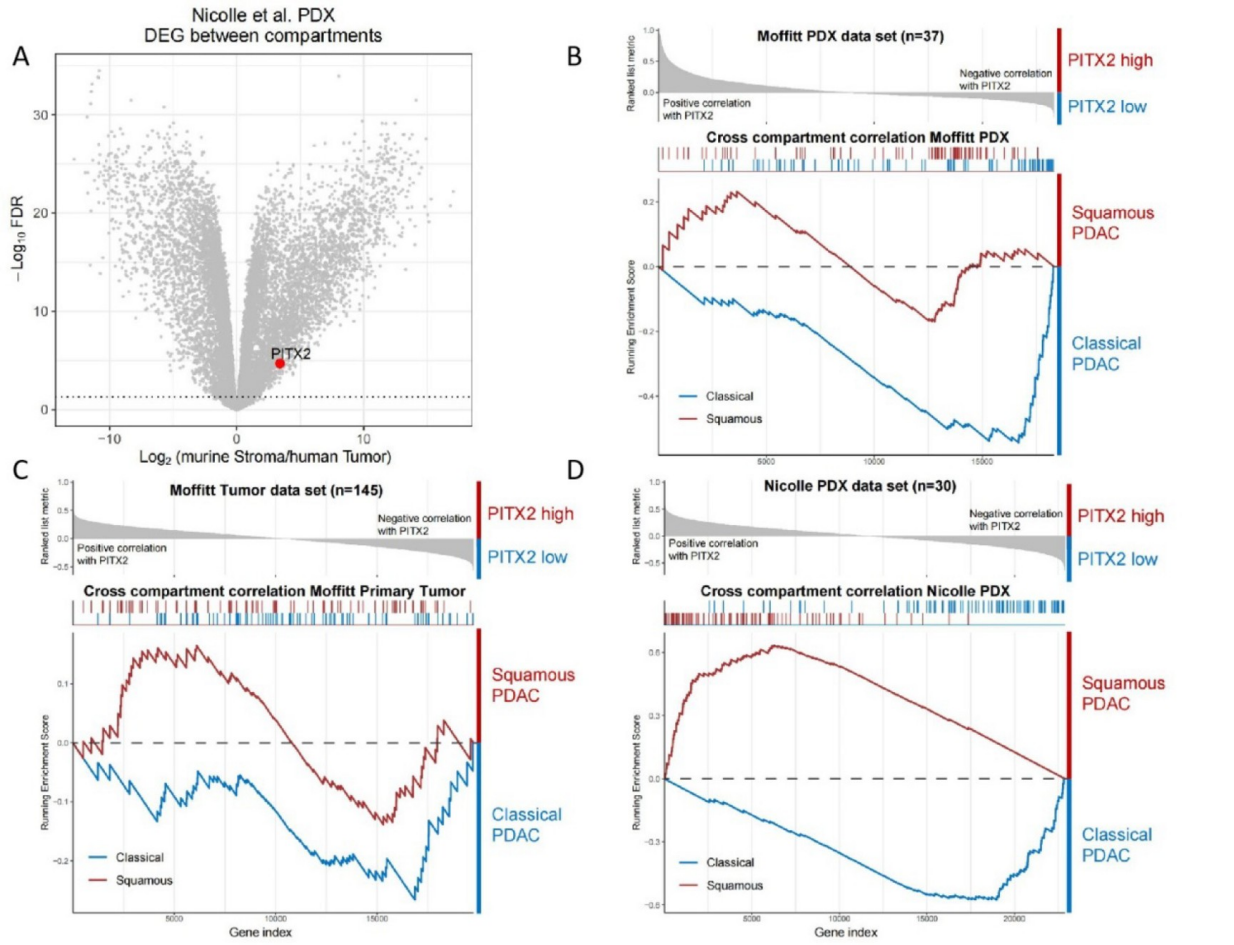
Figure 1 Bioinformatics analysis (A) The volcano plot demonstrates that the PITX2 gene is highly expressed in stromal cells. (B‒D) GSEA suggested that the upregulated expression of PITX2 is related to the squamous type, and the downregulation is related to the classic type in Moffitt PDX and primary tumor or Nicole’s PDX data.

To clarify the distribution of PITX2 in pancreatic cancer, a volcano plot was obtained using bioinformatics data. The results showed that the *PITX2* gene was highly expressed in stromal cells ( Figure 1A). GSEA suggested that the genes significantly positively related to the expression of PITX2 were enriched in squamous-type PDACs, while the genes significantly negatively related to PITX2 were enriched in classic-type PDACs, which also indicated that the upregulated expression of PITX2 was related to the squamous type, and the downregulation was related to the classic type in Moffitt PDX and primary tumor or Nicole’s PDX data ( Figure 1B‒D).

### High PITX2 level in stromal cells is correlated with poor prognosis in PDAC

**Figure 2:**
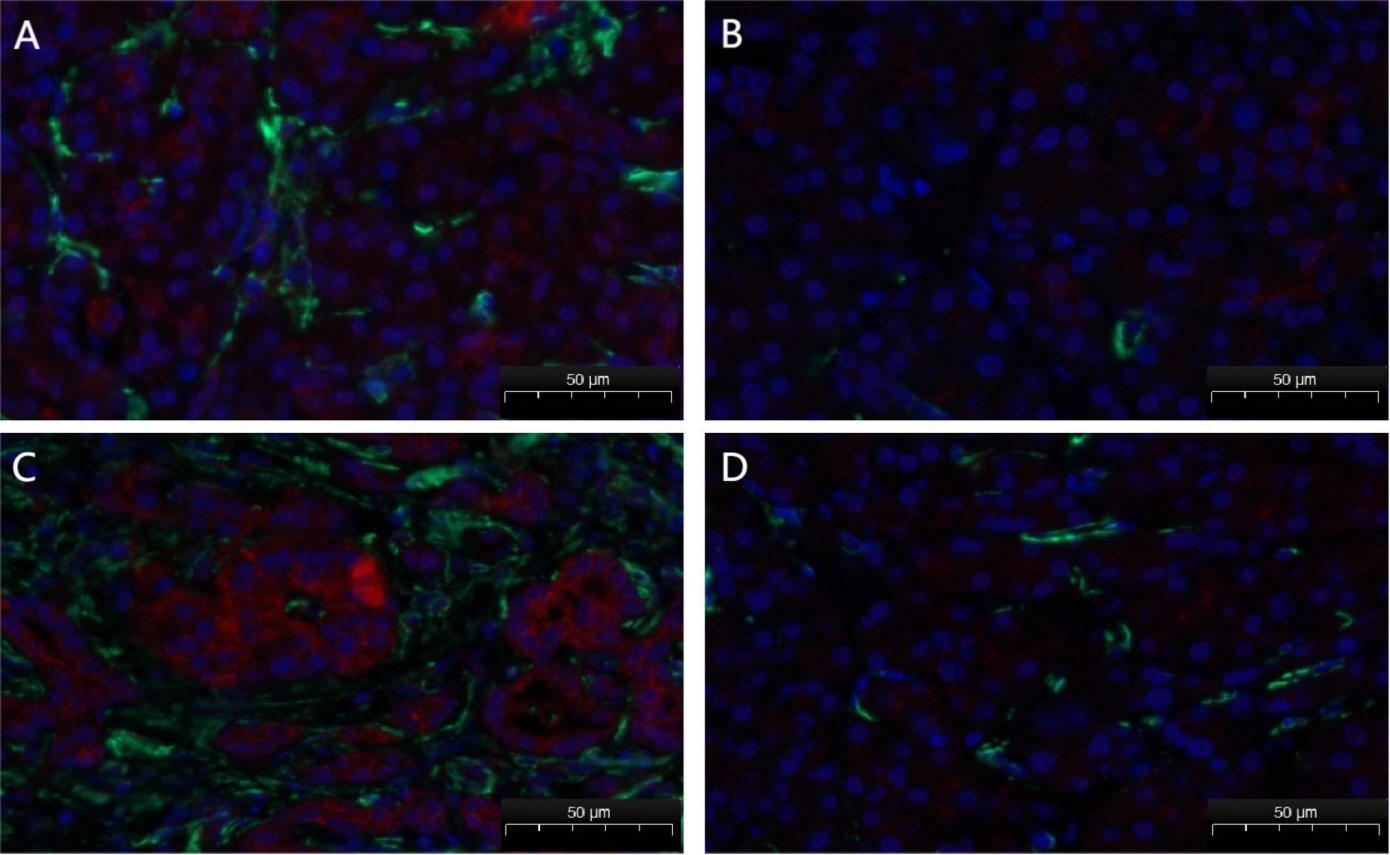
Figure 2 Immunofluorescence staining of tissue microarray PITX2 (red) and α-SMA (green) immunofluorescence of tumor sections. The ratio of PITX2 gene expression in tumor stromal cells (A,C) was higher than that in parenchymal cells (B,D).

**Figure 3:**
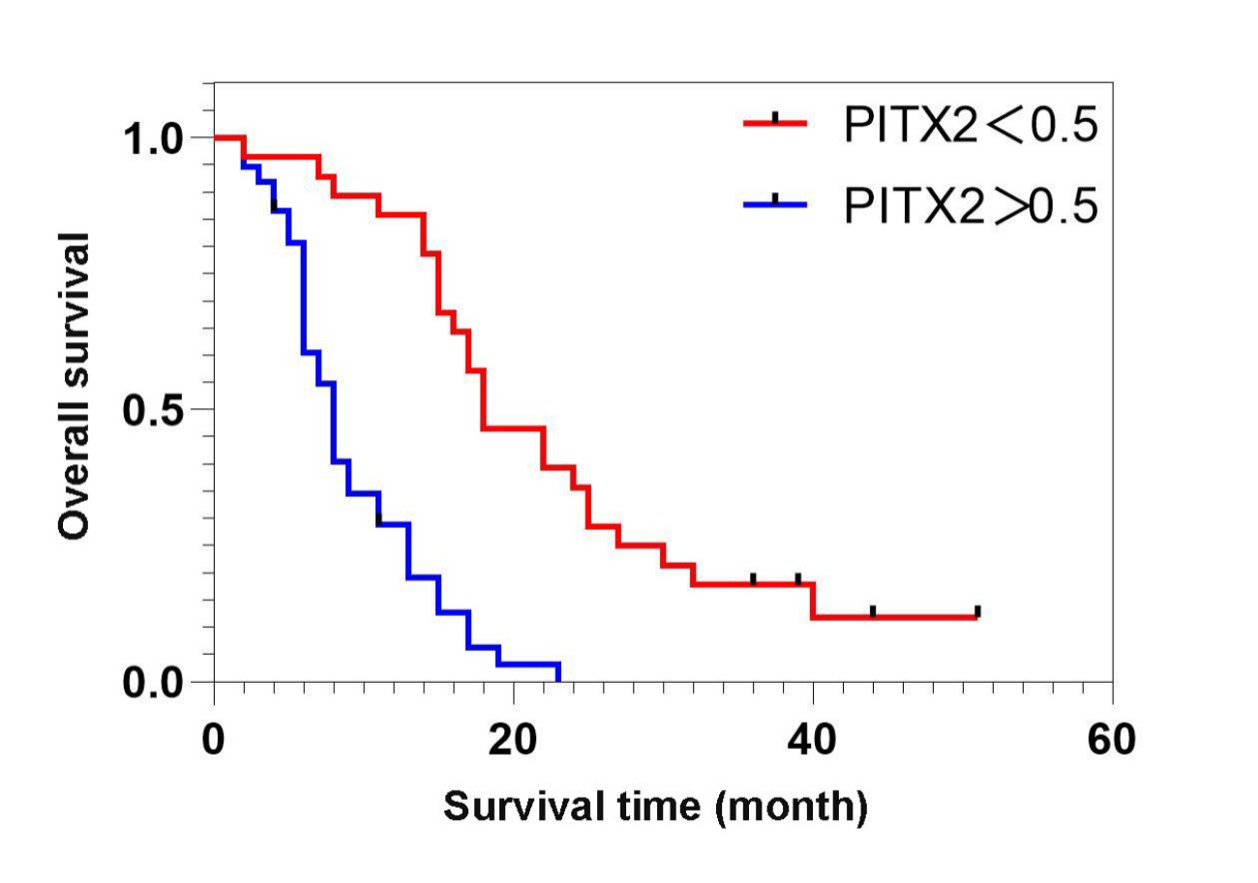
Figure 3 The survival curves were compared using the Kaplan-Meier method according to PITX2 level The red line represents the group with PITX2<0.5, while the blue line represents the group with PITX2>0.5. The log-rank test was performed to evaluate statistical significance.

To further understand the relationship between PITX2 expression in PSCs and the prognosis of PDAC, immunofluorescence staining and tissue microarray analysis were performed. Results showed that the ratio of PITX2 gene expression in tumor stromal cells was higher than that in parenchymal cells (0.525±0.024 vs 0.415±0.031; *P*<0.05; Figure 2). This result is in agreement with that obtained by bioinformatics analysis. The survival curve is shown in Figure 3, which suggests that the prognosis of patients with *PITX2* gene expression higher than 0.5 is poorer than the prognosis of patients with *PITX2* gene expression lower than 0.5 ( *P*<0.05). Univariate analysis and multivariate logistic regression analysis confirmed that among all the factors, tumor staging, distant metastasis, lymph node metastasis, and *PITX2* gene expression higher than 0.5 are possible factors affecting prognosis ( Tables 1 and 2; *P*<0.05).

**Table:** **Table 1** *PITX2* gene expression higher than 0.5 were possible factors affecting prognosis confirmed by univariate analysis

Clinicopathological factor	Number of patients	One year survival rate	Chi square value	*P* value
Tumor location			1.385	0.310
Head	40	0.450		
Body and tail	25	0.600		
CA199			0.337	0.751
Negative	12	0.583		
Positive	53	0.491		
Tumor stage			16.463	0.000
1	9	0.889		
2	9	0.889		
3	21	0.476		
4	26	0.269		
Vascular invasion			0.003	1.000
No	55	0.509		
Yes	10	0.500		
Nerve invasion			0.228	0.783
No	47	0.489		
Yes	18	0.556		
Distant metastasis			16.143	0.000
No	41	0.317		
Yes	24	0.833		
Lymphnode metastasis			15.604	0.000
No	26	0.808		
Yes	39	0.308		
Tumor size			2.011	0.207
<3 cm	39	0.436		
≥3 cm	26	0.615		
PITX2			24.033	0.000
<0.5	28	0.857		
≥0.5	37	0.243

**Table:** **Table 2** Multivariate logistic regression analysis

Clinicopathological factor	B value	Standard error	Wald value	OR	95%CI	*P* value
Tumor stage	‒5.168	2.105	6.026	0.006	0.000–0.353	0.014
Distant metastasis	3.909	1.437	7.396	49.835	2.979–833.598	0.007
Lymphnode metastasis	‒3.676	1.333	7.604	0.025	0.002–0.345	0.006
PITX2>0.5	‒3.006	1.189	6.397	0.049	0.005–0.508	0.011

The data confirmed that among all the factors, tumor staging, distant metastasis, lymph node metastasis, and *PITX2* gene expression higher than 0.5 were possible factors affecting prognosis.

### Isolation, culture and identification of PSCs from fresh pancreatic tumors

**Figure 4:**
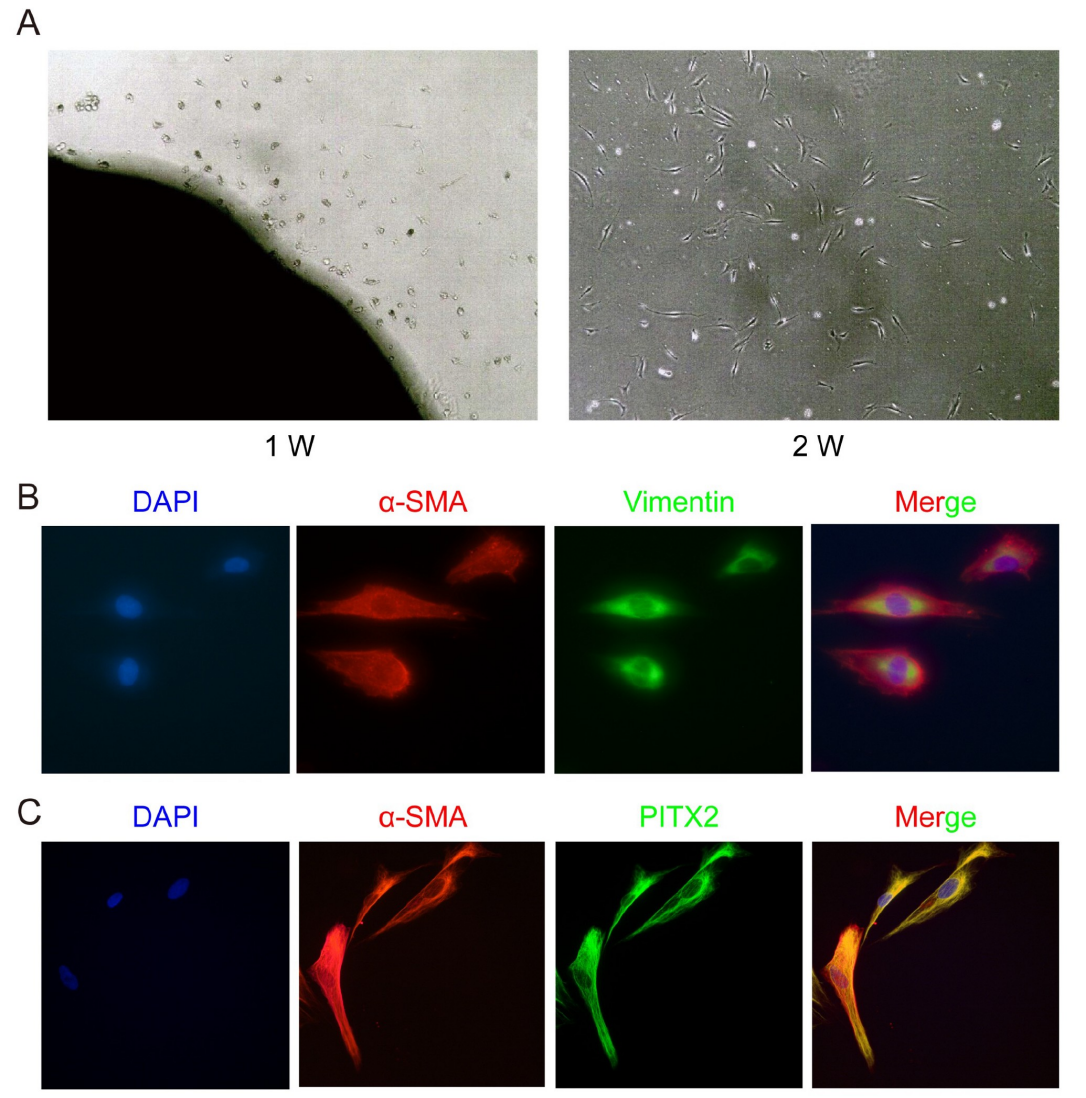
Figure 4 PSC isolation and cellular immunofluorescence (A) PSCs generally grew out of the pancreatic cancer tissue. PSCs were distributed around the tissue at first. After incubation for 2 weeks, PSCs were successfully isolated. (B) Immunofluorescence staining showed the expressions of α-SMA and vimentin, which confirmed the identity of activated PSCs. (C) Immunofluorescence staining showed the positive expression of PITX2 in PSCs.

After incubation for 2 weeks, PSCs were successfully isolated ( Figure 4A). Immunofluorescence staining of α-SMA and vimentin confirmed that the harvested cells were activated PSCs ( Figure 4B), while immunofluorescence staining of PITX2 proved the positive expression of PITX2 in PSCs ( Figure 4C).

### Knockdown of *PITX2* in PSCs inhibits proliferation of PSCs *in vitro* and downregulates the expressions of α-SMA, vimentin, AXIN1, CTNNB1 and LEF1 in PSCs

**Figure 5:**
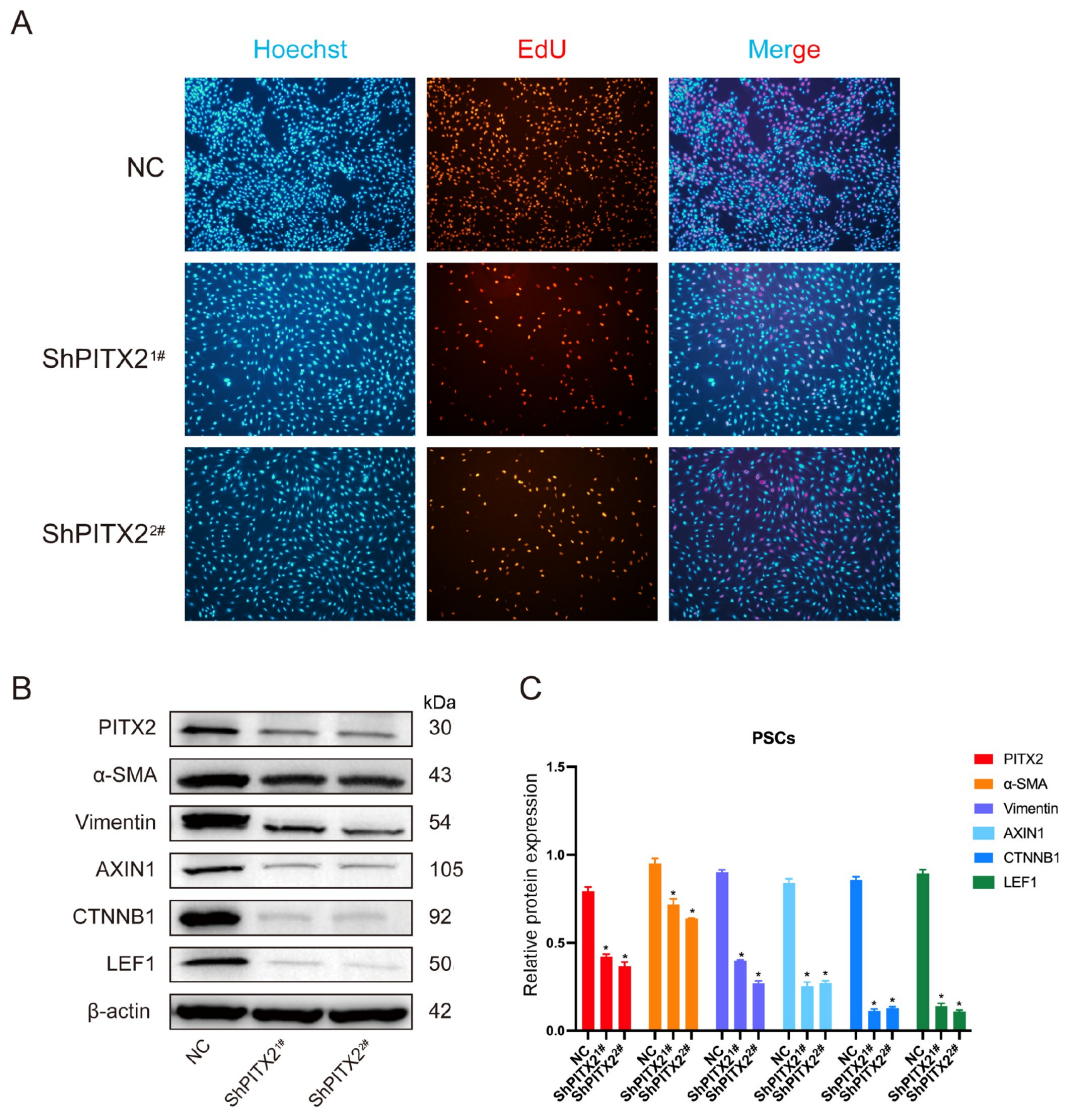
Figure 5 Measurements of the proliferation capacity and related gene expressions of PSCs after knockdown of *PITX2* (A) EdU incorporation assay proved that after knockdown of PITX2, the percentage of cells in S-phase was decreased as well. (B,C) The levels of α-SMA, vimentin, AXIN1, CTNNB1 and LEF1 were decreased in PITX2-knockdown PSCs compared with those in the normal control group.

EdU incorporation assays proved that after knockdown of *PITX2*, the percentage of cells in S-phase was decreased ( Figure 5A). Furthermore, western blot analysis showed that relative protein expressions of α-SMA in NC, ShPITX2 ^1#^ and ShPITX2 ^2#^ were 0.951±0.029, 0.719±0.032 and 0.639±0.002, relative protein expressions of vimentin in NC, ShPITX2 ^1#^ and ShPITX2 ^2#^ were 0.901±0.014, 0.399±0.004 and 0.268±0.014, relative protein expressions of AXIN1 in NC, ShPITX2 ^1#^ and ShPITX2 ^2#^ were 0.840±0.025, 0.253±0.023 and 0.270±0.013, relative protein expressions of CTNNB1 in NC, ShPITX2 ^1#^ and ShPITX2 ^2#^ were 0.858±0.017, 0.112±0.011 and 0.129±0.008, relative protein expressions of LEF1 in NC, ShPITX2 ^1#^ and ShPITX2 ^2#^ were 0.895±0.021, 0.140±0.016 and 0.108±0.008. In all, the levels of α-SMA, vimentin, AXIN1, CTNNB1 and LEF1 were significantly decreased in *PITX2*-knockdown PSCs ( *P* <0.05; Figure 5B,C).

### Knockdown of *PITX2* in PSCs inhibits the proliferation, invasion and migration capacities of pancreatic cancer cells

**Figure 6:**
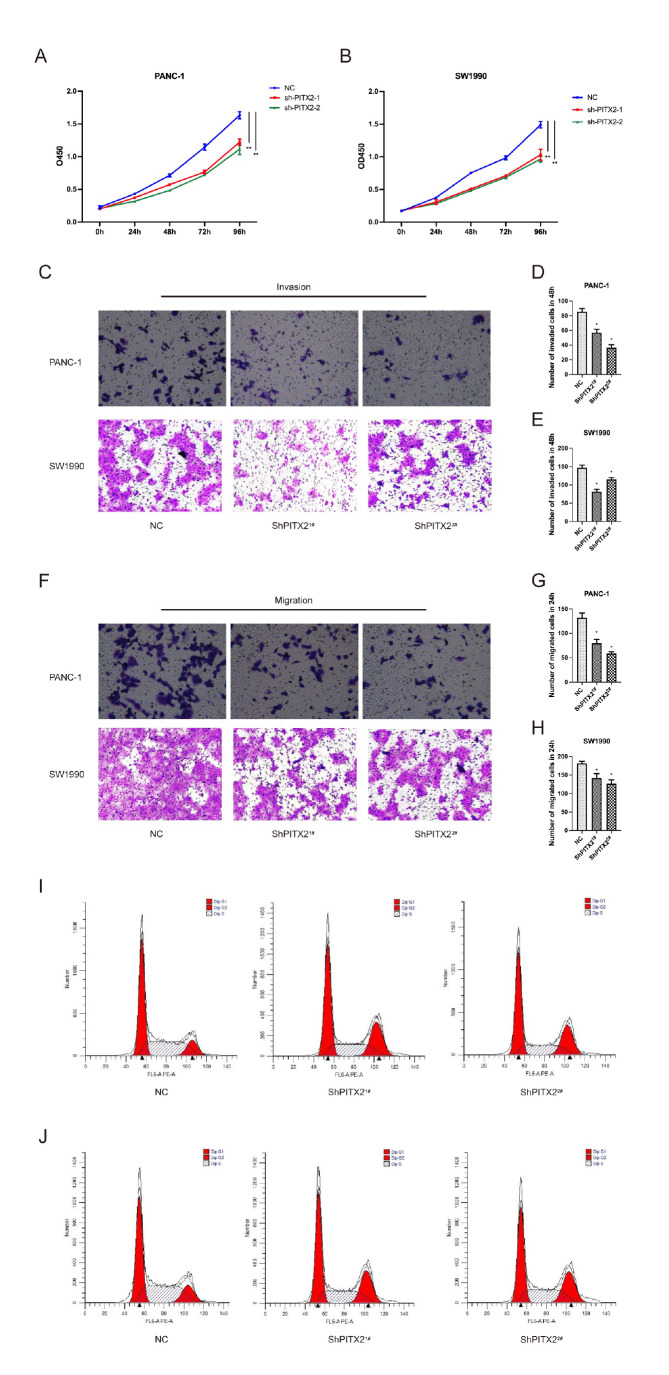
Figure 6 The proliferation, invasion and migration capacities of pancreatic cancer cells after co-cultured with different types of PSCs (A,B) CCK8 assays confirmed that the proliferation of pancreatic cancer cells cocultured with NC-infected PSCs was enhanced compared to that of pancreatic cancer cells cocultured with ShPITX21#- and ShPITX22#-infected PSCs. (C‒H) The invasion (48 h) and migration (24 h) capacities of pancreatic cancer cells cocultured with NC-infected PSCs were significantly greater than those of pancreatic cancer cells cocultured with ShPITX21#- and ShPITX22#-infected PSCs. (I,J) Flow cytometry assay suggested that knockdown of PITX2 in PSCs induced G2/M cell cycle arrest in PANC-1 and SW1990 cells.

To evaluate the proliferation, invasion and migration capacities of pancreatic cancer cells cocultured with PSCs, CCK8, flow cytometry and transwell assays were conducted. The proliferation capacity of pancreatic cancer cells cocultured with NC-infected PSCs was significantly greater than that of pancreatic cancer cells cocultured with ShPITX2 ^1#^- and ShPITX2 ^2#^-infected PSCs ( Figure 6A,B). So were the invasion capacity ( Figure 6C‒E; PANC-1: 85.333±4.509 vs 57±4.583 and 36.333±4.163; SW1990: 146.667±7.638 vs 81.333±6.506 and 115±5.000) and migration capacity ( Figure 6F‒H; PANC-1: 132±9.849 vs 79.667±8.021 and 58.667±3.512; SW1990: 181.667±5.686 vs 142±12.124 and 126.667±10.599). Flow cytometry assays suggested that knockdown of *PITX2* in PSCs induced G2/M cell cycle arrest in pancreatic cancer cells PANC-1 ( Figure 6I) and SW1990 ( Figure 6J).

### Knockdown of *PITX2* in PSCs displays its biological function by deactivating EMT and the Wnt/β-catenin signaling pathway

**Figure 7:**
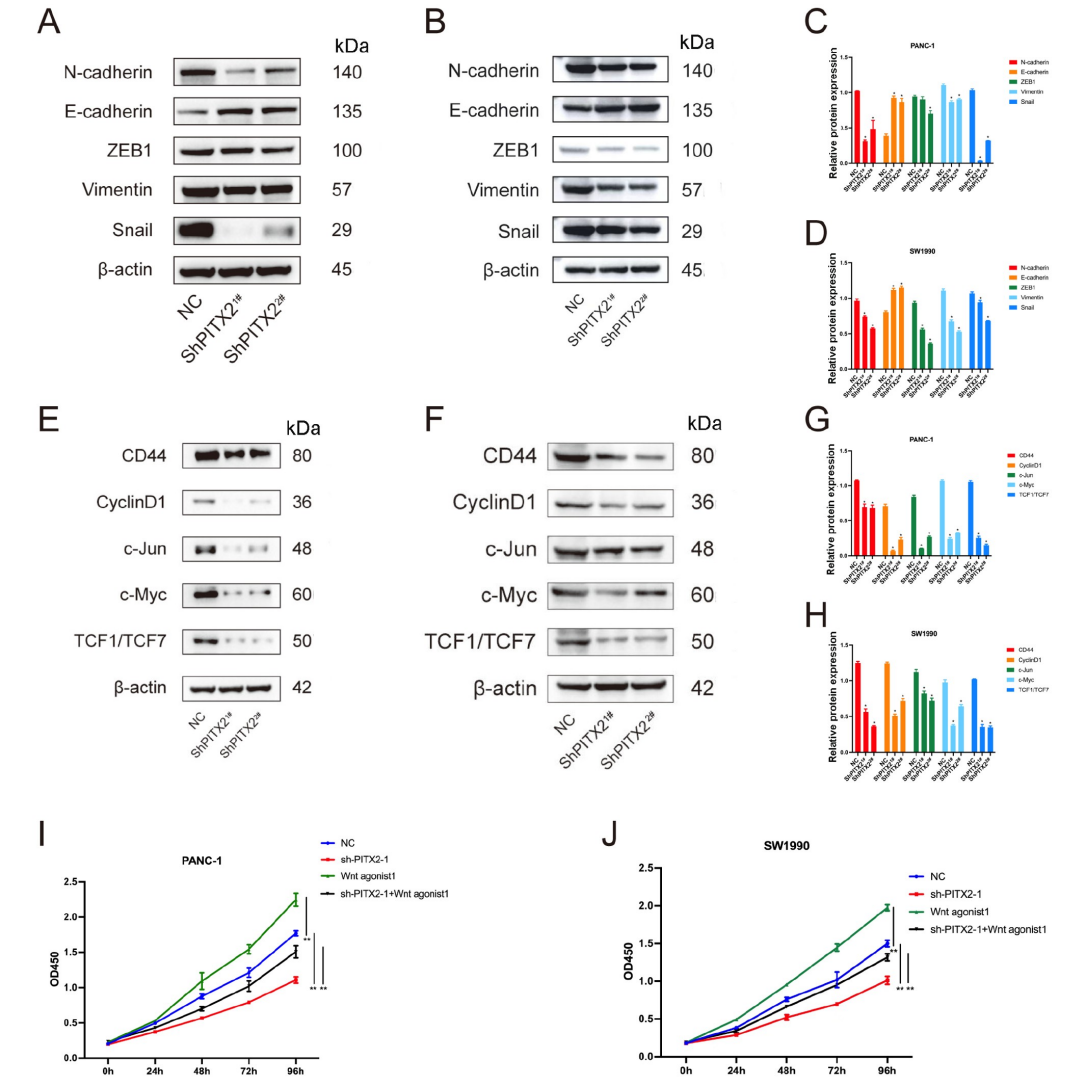
Figure 7 Expressions of the key factors of EMT and Wnt/β-catenin signaling pathway (A,C) Knockdown of PITX2 significantly downregulated the expressions of N-cadherin, vimentin and Snail, while the expression of E-cadherin was upregulated in PANC-1 cells. (B,D) Knockdown of PITX2 significantly downregulated the expressions of N-cadherin, ZEB1, vimentin and Snail, while the expression of E-cadherin was upregulated in SW1990 cells. (E‒H) Knockdown of PITX2 significantly downregulated the expressions of CD44, Cyclin D1, c-Jun, c-Myc and TCF1/TCF7 in the Wnt/β-catenin signaling pathway. (I,J) Wnt/β-catenin agonist 1 rescued the effect of PITX2 on cell proliferation.

Previous studies have reported that EMT and the Wnt/β-catenin pathway are regulated by PITX2 in many diseases [ 11, 18‒ 20 ]. We performed experiments to test the influence of PITX2 in PSCs on EMT and Wnt/β-catenin downstream genes in pancreatic cancer cells. In PANC-1 cells, knockdown of *PITX2* significantly downregulated the expressions of N-cadherin, vimentin and Snail, while the expression of E-cadherin was upregulated ( Figure 7A,C). In SW1990 cells, knockdown of *PITX2* significantly downregulated the expressions of N-cadherin, ZEB1, vimentin and Snail, while the expression of E-cadherin was upregulated ( Figure 7B,D). Knockdown of *PITX2* significantly downregulated the expressions of CD44, CyclinD1, c-Jun, c-Myc and TCF1/TCF7 in the Wnt/β-catenin signaling pathway ( Figure 7E‒H). Wnt/β-catenin agonist 1, an activator of the Wnt/β-catenin pathway, rescued the effect of PITX2 on cell proliferation ( Figure 7 I,J).

## Discussion

*PITX* genes are evolutionarily conserved and have been discovered in almost all members of the animal kingdom, from the simplest animal species of Placozoa to humans [21]. PITX2 is essential for the development of the oral cavity and abdominal wall and regulates the formation and symmetry of other organs, including the pituitary, heart, gut, and lung, by controlling growth control genes upon activation of the Wnt/β-catenin signaling pathway [6]. The involvement of the *PITX2* gene in tumorigenesis has shaped its use as a diagnostic marker and potential drug target [6]. Previous studies reported that elevated expression of PITX2 is associated with advanced progression and poor prognosis of lung adenocarcinoma [ 7, 8], colorectal cancer [ 9, 10], ovarian cancer [11], esophageal squamous cell carcinoma [12] and thyroid cancer [ 13, 14].

In the present study, bioinformatics analysis and tissue microarray analysis showed that *PITX2* gene expression in tumor stromal cells is higher than that in parenchymal cells among pancreatic cancer patients. The prognosis of patients with *PITX2* gene expression higher than 0.5 was poorer than the prognosis of patients with *PITX2* gene expression lower than 0.5. Univariate analysis and multivariate logistic regression analysis also demonstrated that *PITX2* gene expression higher than 0.5 is a possible factor affecting prognosis.

The microenvironment of pancreatic cancer plays an important role in the progression of pancreatic cancer. The most important shaper of the pancreatic cancer microenvironment is PSCs [22]. Activated PSCs generate a variety of cytokines and growth factors and exert important biological functions to maintain pancreatic cancer progression, such as proliferation, metastasis, immunoregulation and chemotherapy resistance [23].

Given that *PITX2* gene expression in tumor stromal cells is higher than that in parenchymal cells among pancreatic cancer patients and that *PITX2* gene expression in tumor stromal cells higher than 0.5 is a factor affecting prognosis, we hypothesized that the upregulation of PITX2 in PSCs may be associated with poor differentiation and poor prognosis, while the downregulation of PITX2 may function conversely. Thus, we isolated primary human PSCs from resected pancreatic tumors. Cellular immunofluorescence confirmed the positive staining of α-SMA and vimentin. Previous studies reported that α-SMA immunostaining indicates the activation of PSCs [ 22, 24]. Furthermore, we induced stable knockdown of *PITX2* in PSCs through lentiviral infection. In our study, after stable knockdown of PITX2, PSCs showed inhibited proliferation. Knockdown of *PITX2* inhibited the expressions of the mesenchymal marker vimentin and the ECM protein a-SMA, which confirmed the deactivation of PSCs upon lentiviral infection. A previous study showed that in the String database, PITX2 was predicted to interact with LEF1, CTNNB1 and AXIN, all of which are key factors of the Wnt/β-catenin signaling pathway [8]. Therefore, the expressions of LEF1, CTNNB1 and AXIN1 were evaluated. The results showed that *PITX2*-knockdown PSCs had lower expressions of LEF1, CTNNB1 and AXIN1 than normal control PSCs.

Upon coculture with pancreatic cancer cells, knockdown of the *PITX2* gene in PSCs inhibited the growth, migration and invasion of pancreatic cancer cells. Previous studies have reported that EMT and the Wnt/β-catenin pathway are regulated by PITX2 in many diseases [ 11, 18‒ 20]. The results that knockdown of *PITX2* inhibited the expressions of the mesenchymal marker vimentin and ECM protein a-SMA, as well as key factors of the Wnt/β-catenin signaling pathway, including LEF1, CTNNB1 and AXIN1, in PSCs shed light on the underlying mechanism of the inhibited progression of pancreatic cancer cells. We performed experiments to test the influence of stable knockdown of *PITX2* in PSCs on EMT and Wnt/β-catenin downstream genes in pancreatic cancer cells. The results were as expected.

However, there were still some limitations in our study. PSCs consist of most of the CAFs in PDAC. The present study confirmed the existence of a relationship between PITX2 in PSCs and pancreatic cancer cells. However, how it works remains unknown. Studies on how PITX2 in PSCs affects the proliferation, invasion and migration of pancreatic cancer cells are being carried out.

In conclusion, *PITX2* knockdown in PSCs inhibited the growth, migration and invasion of pancreatic cancer cells via reduced EMT and Wnt/β-catenin signaling.
